# Supplementary material for: Content-rich biological network constructed by mining PubMed abstracts
Source: BMC Bioinformatics. 2004 Oct 8;5:147. doi: 10.1186/1471-2105-5-147 (PMC528731; doi:10.1186/1471-2105-5-147)
Supplement: Additional File 2 — The original results of the above study (non-essential files are deleted to keep the file size under the limit set by BMC bioinformatics). [file 1471-2105-5-147-S2.bz2 › chilibotAdditionalFile2/dip10/612ID7791872E10995/html/RAF1_RAP1A.html]

 


 **RAF1** and **RAP1A** 
  
Found 29 abstracts in PubMed, retrieved 10.  
 

 What does Google say? 
 PDF only 
| .edu only 

---

**Interactive relationship** (e.g. stimulation, inhibition, etc)

**Neutral relationship**- Finally, a B Raf  [ **RAF1** ]  CRD mutant whose interaction with  **Rap1A**  is selectively enhanced was isolated and found to possess the double mutation K252E M278T.  Ref: 10454553 Mol Cell Biol, 1999
- Using the selective interactions of Ras and Krev 1  [ **RAP1A** ]   **Rap1A**  with Raf  [ **RAF1** ] , RalGDS, and Krit1 as a model, we systematically compared LexA and cI fused baits and reporters.  Ref: 10358061 J Biol Chem, 1999
- ... B Raf  [ **RAF1** ] , whose CRD is replaced by Raf 1  [ **RAF1** ]  CRD, lost its response to  **Rap1A** .  Ref: 10454553 Mol Cell Biol, 1999
- The Ras homologue  **Rap1A**  is incapable of activating Raf 1  [ **RAF1** ]  and even antagonizes Ras dependent activation of Raf 1  [ **RAF1** ] .  Ref: 10454553 Mol Cell Biol, 1999
- The importance of the interaction at the CRD is further supported by a domain shuffling experiment between Raf 1  [ **RAF1** ]  and B Raf  [ **RAF1** ] , which clearly indicated that the nature of CRD determines the specificity of response to  **Rap1A**  Raf 1  [ **RAF1** ] , whose CRD is replaced by B Raf  [ **RAF1** ]  CRD, became activatable by  **Rap1A** .  Ref: 10454553 Mol Cell Biol, 1999
- On the other hand, B Raf  [ **RAF1** ] , another Raf  [ **RAF1** ]  family member, is activatable by both Ras and  **Rap1A** .  Ref: 10454553 Mol Cell Biol, 1999
- These results indicate that the strength of interaction with Ras and  **Rap1A**  at its CRD may be a critical determinant of regulation of the Raf  [ **RAF1** ]  kinase activity by the Ras family small GTPases.  Ref: 10454553 Mol Cell Biol, 1999
- The complex of Ras with the Ras binding domain RBD of Raf  [ **RAF1** ]  has been modelled by homology to the E30D, K31E  **Rap1A**  RBD complex, and both have been subjected to multiple molecular dynamics simulations in solution.  Ref: 10090289 Proteins, 1999
- The effect of  **Rap1A**  V12 was dramatically potentiated by the concurrent activation of PKC, the cAMP pathway, and Ras, and this potentiation was blocked by dominant negative mutants of Ras and Raf  [ **RAF1** ] .  Ref: 12473665 J Biol Chem, 2003
- We proposed previously that this property of  **Rap1A**  may be attributable to its greatly enhanced interaction with Raf 1  [ **RAF1** ]  CRD compared to Ras.  Ref: 10454553 Mol Cell Biol, 1999

**Non-interactive relationship** (e.g. studied together, co-existance, homology, etc.)

- B Raf  [ **RAF1** ]  carrying this mutation was not activated by  **Rap1A**  but retained its response to Ras.  Ref: 10454553 Mol Cell Biol, 1999
- In cells expressing the activated  **Rap1A**  protein, a PI3K Raf  [ **RAF1** ]  MEK dependent pathway mediates transient ERK activation.  Ref: 11146560 Oncogene, 2000
- When interactions with Ras and  **Rap1A**  were measured, B Raf  [ **RAF1** ]  CRD did not exhibit the enhanced interaction with  **Rap1A** , suggesting that the strength of interaction at CRDs may account for the differential action of  **Rap1A**  on Raf 1  [ **RAF1** ]  and B Raf  [ **RAF1** ] .  Ref: 10454553 Mol Cell Biol, 1999
